# Impact of tobacco-related chronic obstructive pulmonary disease on developmental trajectories of comorbidities in the Taiwan population

**DOI:** 10.1038/s41598-020-78325-y

**Published:** 2020-12-03

**Authors:** Te-Wei Ho, Yi-Ju Tsai, Chun-Ta Huang, Angela Shin-Yu Lien, Feipei Lai

**Affiliations:** 1 Graduate Institute of Biomedical Electronics and Bioinformatics, College of Electrical Engineering and Computer Science, National Taiwan University, Taipei, Taiwan; 2 Department of Surgery, College of Medicine, National Taiwan University, Taipei, Taiwan; 3 Graduate Institute of Biomedical and Pharmaceutical Science, College of Medicine, Fu Jen Catholic University, New Taipei City, Taiwan; 4 Department of Internal Medicine, National Taiwan University Hospital, No. 7, Chung-Shan South Road, Taipei, 100 Taiwan; 5 Graduate Institute of Clinical Medicine, National Taiwan University, Taipei, Taiwan; 6 School of Nursing, College of Medicine, Chang Gung University, Taoyuan City, Taiwan; 7 Division of Endocrinology and Metabolism, Department of Internal Medicine, Chang Gung Memorial Hospital Linkou Branch, Taoyuan City, Taiwan

**Keywords:** Chronic obstructive pulmonary disease, Comorbidities

## Abstract

Comorbidities adversely affect the quality of life and survival of patients with chronic obstructive pulmonary disease (COPD), and timely identification and management of comorbidities are important in caring for COPD patients. This study aimed to investigate the impact of COPD on long-term developmental trajectories of its comorbidities. From 2010 to 2013, all spirometry-confirmed COPD patients with a 5-year follow-up period were identified as the cases. The prevalence of comorbidities and their trajectories in COPD cases were obtained and compared with those in non-COPD controls matched for age, sex, smoking status and Charlson comorbidity index (CCI). Over the study period, a total of 682 patients, 341 each in COPD and control groups were included, with a mean age of 69.1 years and 89% male. The baseline mean CCI was 1.9 for both groups of patients and significantly increased to 3.4 and 2.7 in COPD and control groups after 5 years, respectively (both P < 0.001). Through the 5-year follow-up, a significant increase in the prevalence of all comorbidities of interest was observed in the COPD cohort and the incidence was remarkably higher for hypertension [incidence rate ratio (IRR) 1.495; 95% confidence interval (CI) 1.017–2.198], malignancy (IRR 2.397; 95% CI 1.408–4.081), diabetes mellitus (IRR 2.927; 95% CI 1.612–5.318), heart failure (IRR 2.531; 95% CI 1.502–4.265) and peptic ulcer disease (IRR 2.073; 95% CI 1.176–3.654) as compared to the non-COPD matched controls. In conclusion, our findings suggest that the presence of COPD may be considered a pathogenic factor involved in the development of certain comorbidities.

## Introduction

Chronic obstructive pulmonary disease (COPD), featured by persistent airflow limitation and respiratory symptoms, is one of the most important global health threats and the third leading cause of death worldwide^1^. Compared to non-COPD individuals, patients with COPD are more likely to suffer from comorbidities, such as cardiovascular disease and metabolic syndrome^2–5^. The increased prevalence may be attributable to shared risk factors (e.g., smoking) or pathophysiological mechanisms (e.g., chronic inflammation)^1,3^. Comorbidities are of paramount importance since it is well known that some of them, such as heart failure, coronary artery disease and diabetes mellitus, have a significant impact on health status and health care utilization and increase the risks of hospitalization and mortality in COPD patients^2,6–9^.

It has been suggested that comorbidities should be routinely looked for and appropriately treated in any patient with COPD as per clinical practice guideline^1^, although the evidence to support this contention and practical recommendations are still lacking. Over the past 20 years, a barrage of publications have delineated a clearer picture of the association between COPD and its comorbidities^2–4,6–8^; however, little if any is known about the trajectories in the comorbidity burden along the clinical course of patients with COPD. In this regard, it would be valuable to better understand this issue because the messages can inform health decision-making and guideline development.

In this study, we therefore investigated the prevalence of comorbidities in spirometry-confirmed COPD patients and observed their evolution through the 5-year follow-up period. Meanwhile, a matched non-COPD cohort was assembled to determine the impact of COPD on the developmental trajectories of those comorbidities.

## Methods

### Study design and settings

This long-term retrospective cohort study was conducted at the National Taiwan University Hospital, a tertiary-care referral center in Northern Taiwan. In our institution, patients with COPD were in principle assessed and treated based on the contemporary Global Initiative for Chronic Obstructive Lung Disease (GOLD) guidelines^10,11^. The Research Ethics Committee of the National Taiwan University Hospital has approved the study protocol and waived the informed consent because of descriptive and retrospective nature of the study.

### Study population

**Figure 1:**
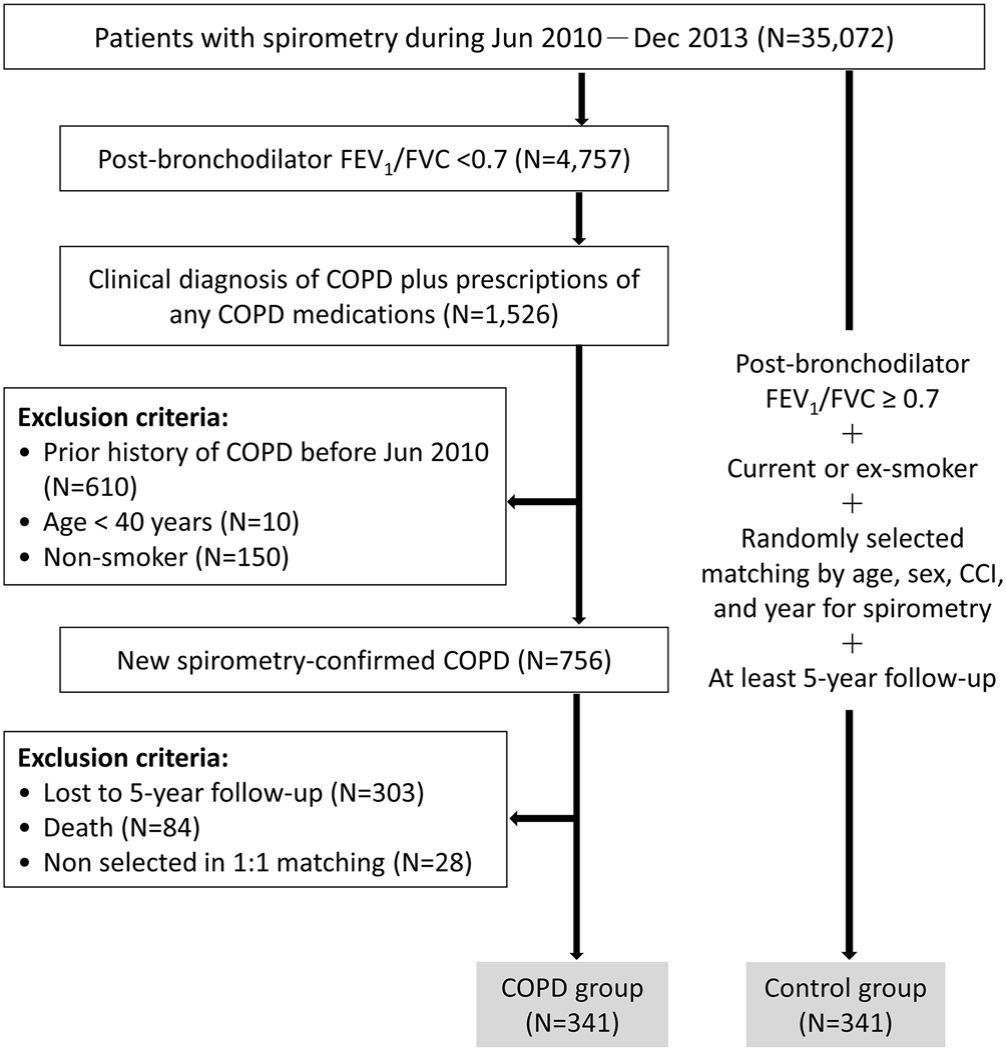
Study flow diagram. *CCI* Charlson comorbidity index, *COPD* chronic obstructive pulmonary disease, *FEV*_*1*_ forced expiratory volume in 1 s, *FVC* forced vital capacity, *GOLD* global initiative for chronic obstructive lung disease.

To assemble the COPD cohort with a 5-year follow-up period for evaluating the evolution of the comorbidities, all patients having a spirometry test from June 2010 to December 2013 were screened for eligibility in this study (Fig. 1). Inclusion criteria included: (a) persistent airflow limitation, defined as a proportion of the forced vital capacity exhaled in the first second (FEV_1_/FVC) < 0.7 after administration of a bronchodilator; (b) a clinical diagnosis of COPD plus a prescription of any COPD medications, including β_2_-agonists, anticholinergics and methylxanthines; and (c) having follow-up for COPD prescriptions for at least 5 consecutive years. The date of COPD diagnosis was defined as the index date. Patients were excluded if they had any of the following: (a) prior history of COPD before June 2010; (b) age < 40 years; (c) non-smokers; (d) loss of follow-up or death within 5 years of the index date.

To compare the change in the comorbidity burden between COPD and non-COPD patients, all subjects with a spirometry test showing post-bronchodilator FEV_1_/FVC ≥ 0.7 and a current or former smoking status were randomly selected to assemble the non-COPD cohort after matching for age, sex, Charlson comorbidity index (CCI) and year for spirometry. A minimal 5-year follow-up period after the date of spirometry was also a prerequisite for the controls.

### Data collection

Data retrieved at baseline were age, sex, body mass index, smoking status, comorbidities needed to calculate the CCI and FEV_1_% of the predicted value. During the follow-up period, messages regarding the development of comorbidities of interest was pursued. Through literature review, we included hypertension, coronary artery disease, malignancy, diabetes mellitus, hyperlipidemia, heart failure, peptic ulcer disease, peripheral arterial disease, atrial fibrillation and liver cirrhosis as the comorbidities of interest since those comorbidities have been shown to be prognostically detrimental among the COPD patients^1,8,12^. Meanwhile, similar information was obtained from the control subjects at baseline and during follow-up. To obtain the aforementioned information, patient medical records were reviewed in detail. The presence and development of all the comorbidities of interest needed to be affirmed by both the clinician's diagnosis and objective criteria for the diseases^13–20^.

### Endpoints

Outcomes of interest in this study were the baseline prevalence of medical record notes of the above-specified comorbidities and their incidence along the 5-year follow-up in the COPD patients. We also sought to realize the impact of COPD on comorbidity trajectories by comparing to non-COPD controls.

### Statistical analysis

Categorical variables were presented as number (%) and continuous variables were displayed as mean ± standard deviation. Comparisons of variables between COPD patients and controls were performed using the chi-square or Student’s t-test, as appropriate. The P for trend was used to evaluate the trend of comorbidity prevalence along the follow-up time. Repeated measures analysis of variance was conducted to compare the trend of change in the prevalence of comorbidities between the COPD and control cohorts. The 5-year incidence rate ratios (IRRs) with their 95% confidence intervals (CIs) for each comorbidity of interest were also calculated. The multivariate logistic regression model was constructed to identify independent risk factors associated with the development of two or more comorbidities of interest over the 5-year follow-up and odds ratios (ORs) with 95% CIs were reported. Data analysis was performed using the SPSS software (Version 20.0; IBM Corp., Armonk, NY, US). A two-tailed P value of < 0.05 was deemed statistically significant.

### Ethics declarations

The study protocol was approved by the Research Ethics Committee of the National Taiwan University Hospital and informed consent was waived because of the retrospective nature of the study.

## Results

### Baseline characteristics of patients

A total of 756 patients newly diagnosed with spirometry-confirmed COPD were identified between June 2010 and December 2013 (Fig. 1). Of those, 369 subjects had at least 5-year follow-up period from the index date of COPD diagnosis to evaluate the long-term evolution of their comorbidities. After the matching process, the study sample included 341 COPD patients and an equal number of matched non-COPD individuals. Table 1 shows the characteristics of the study cohort. The mean age of COPD patients was 69.4 years and 89% were males. The average FEV_1_% of the predicted value for patients with COPD was 0.70 ± 0.23 and the majority (N = 277, 81%) of those were categorized as GOLD stage 1 or 2. Compared to the COPD cohort, non-COPD controls were more like to be overweight (55% vs. 42%).

**Table 1:** Baseline characteristics of the patients with chronic obstructive pulmonary disease and their matched controls.

Characteristic	Non-COPD controls	Patients with COPD	P value
No. of patients	N = 341	N = 341	
Age, years	68.9 ± 10.3	69.4 ± 9.9	0.515
Male sex	304 (89)	304 (89)	1.000
**Smoking status**			
Current smoker	182 (53)	190 (56)	0.538
Ex-smoker	159 (47)	151 (44)	
**Body mass index, kg/m**^2^	24.7 ± 4.5	23.4 ± 4.0	< 0.001
< 18.5	16 (5)	31 (9)	0.001
18.5–24	132 (40)	161 (48)	
> 24	184 (55)	141 (42)	
FEV_1_, % predicted	103 ± 20	70 ± 23	< 0.001
**GOLD stage**			
1		117 (34)	
2		160 (47)	
3		50 (15)	
4		14 (4)	

*COPD* chronic obstructive pulmonary disease, *FEV*_*1*_ forced expiratory volume in 1 s, *GOLD* global initiative for chronic obstructive lung disease, *N/A* not applicable.

### Burden of comorbidities

In terms of comorbidity burden, the baseline CCI was the same for both groups of patients; however, a significant difference in the prevalence of certain comorbidities of interest was observed (Table 2). At baseline, a higher proportion of non-COPD controls had comorbid hypertension (43% vs. 29%), malignancy (24% vs. 16%), diabetes mellitus (26% vs. 16%), hyperlipidemia (20% vs. 11%) and peptic ulcer disease (14% vs. 7%) than did COPD patients.

**Table 2:** Burden of comorbidities in patients with chronic obstructive pulmonary disease and the matched controls.

Characteristics	Non-COPD controls	Patients with COPD	P value
	N = 341	N = 341	
Charlson comorbidity index	1.9 ± 1.3	1.9 ± 1.3	1.000
**Comorbidity**			
Hypertension	148 (43)	99 (29)	< 0.001
Coronary artery disease	84 (25)	73 (21)	0.317
Malignancy	82 (24)	55 (16)	0.010
Diabetes mellitus	87 (26)	54 (16)	0.002
Hyperlipidemia	67 (20)	36 (11)	0.001
Heart failure	32 (10)	29 (9)	0.687
Peptic ulcer disease	49 (14)	24 (7)	0.002
Peripheral arterial disease	19 (6)	13 (4)	0.277
Atrial fibrillation	15 (4)	8 (2)	0.138
Liver cirrhosis	6 (2)	3 (1)	0.505

*COPD* chronic obstructive pulmonary disease, *FEV*_*1*_ forced expiratory volume in 1 s, *GOLD* global initiative for chronic obstructive lung disease, *N/A* not applicable.

### Comorbidity evolution

**Figure 2:**
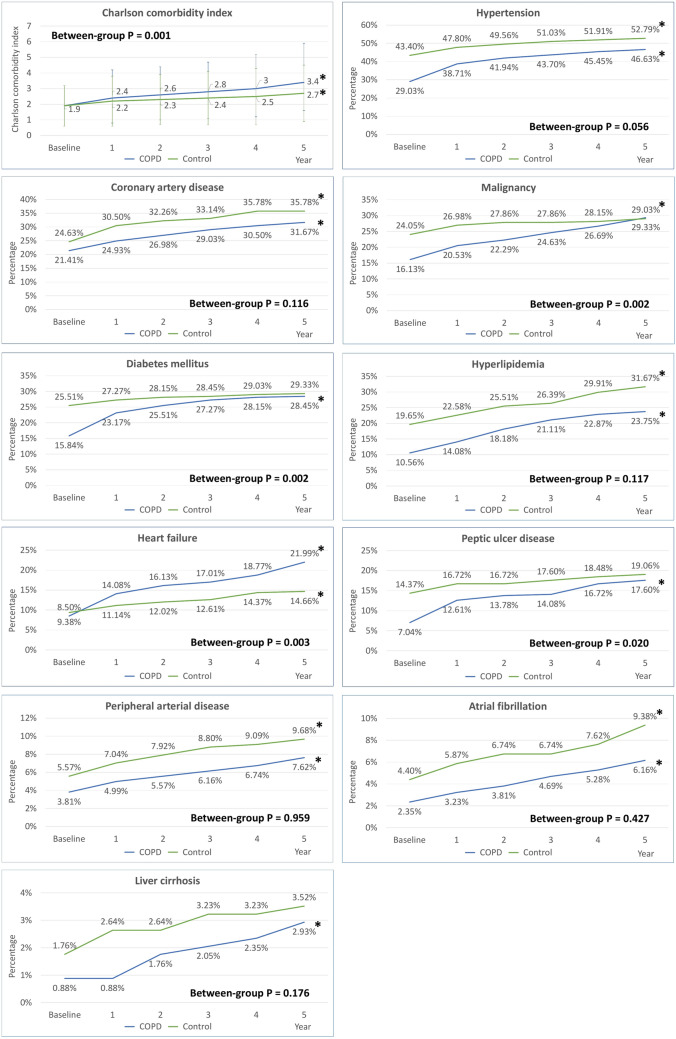
Trajectories of Charlson comorbidity index and prevalence of comorbidities among COPD patients and matched controls. *P for trend < 0.05. *COPD* chronic obstructive pulmonary disease.

Figure 2 displays the developmental trajectories of the CCI and comorbidities of interest in COPD and matched control patients. Over the 5-year follow-up period, a significant increase in the CCI was observed in both COPD patients and controls, but the trend was more prominent in patients with COPD (between-group P = 0.001). Hypertension, coronary artery disease, malignancy, diabetes mellitus and hyperlipidemia were the leading comorbidities of interest for both groups of patients on enrollment. Through the subsequent 5 years, there was a significantly increasing trend in the prevalence of all those comorbidities among the COPD patients; however, the increasing trend was only remarkable for hypertension, coronary artery disease and hyperlipidemia in the control patients. With regards to less prevalent comorbidities, namely, heart failure and peptic ulcer disease, a dissimilarity in the trend of increasing prevalence was also found between two patient groups, with more prominent change in the COPD patients.

For the annual incidence, hypertension (3.5%), heart failure (2.7%), hyperlipidemia (2.6%), malignancy (2.6%) and diabetes mellitus (2.5%) were the top five comorbidities in the COPD cohort during the 5-year follow-up (Fig. 2). Table 3 shows the 5-year IRRs of comorbidities between COPD and non-COPD matched control patients. Significant differences between groups were seen in diabetes mellitus (IRR 2.927; 95% CI 1.612–5.318), heart failure (IRR 2.531; 95% CI 1.502–4.265), malignancy (IRR 2.397; 95% CI 1.408–4.081), peptic ulcer disease (IRR 2.073; 95% CI 1.176–3.654) and hypertension (IRR 1.495; 95% CI 1.017–2.198).

**Table 3:** Five-year incidence rate ratio and 95% confidence interval on comorbidities between patients with chronic obstructive pulmonary disease and control subjects.

Comorbidities	COPD vs. control		P value
	IRR	95% CI	
Hypertension	1.495	1.017–2.198	0.040
Coronary artery disease	0.883	0.577–1.353	0.568
Malignancy	2.397	1.408–4.081	0.001
Diabetes mellitus	2.927	1.612–5.318	< 0.001
Hyperlipidemia	0.986	0.667–1.457	0.944
Heart failure	2.531	1.502–4.265	< 0.001
Peptic ulcer disease	2.073	1.176–3.654	0.012
Peripheral arterial disease	0.912	0.435–1.909	0.806
Atrial fibrillation	0.749	0.370–1.516	0.421
Liver cirrhosis	1.156	0.393–3.405	0.792

*CI* confidence interval, *COPD* chronic obstructive pulmonary disease, *IRR* incidence rate ratio.

### Risk factors with comorbidity development

Through the 5-year follow-up, approximately one-fourths (N = 157, 23%) of the total patient cohort developed two or more comorbidities of interest. Using multivariate logistic regression analysis (Table 4), taking into account patients’ baseline characteristics, i.e., a diagnosis of COPD, age, sex, body mass index and smoking status, we identified the presence of COPD (OR 1.527; 95% CI 1.054–2.212), age ≥ 65 years (OR 1.651; 95% CI 1.101–2.475) and current smoker (OR 1.675; 95% CI 1.150–2.439) as the independent risk factors associated with the development of two or more comorbidities.

**Table 4:** Multivariate logistic regression showing baseline features associated with the development of two or more comorbidities of interest across 5 years.

Parameters	Odds ratio	95% CI	P value
Chronic obstructive pulmonary disease	1.527	1.054–2.212	0.025
Age, ≥ 65 years	1.651	1.101–2.475	0.015
**Body mass index, kg/m**^**2**^			
< 18.5	Reference		
18.5–24	0.745	0.353–1.569	0.438
> 24	1.452	0.699–3.020	0.318
**Smoking status**			
Ex-smoker	Reference		
Current smoker	1.675	1.150–2.439	0.007

*CI* confidence interval.

## Discussion

To the best of our knowledge, this is the first study to elaborate the developmental trajectories of the comorbidity burden through a long 5-year follow-up in patients with COPD. We found a significant increasing trend in the prevalence of all those comorbidities of interest in COPD patients. Compared to non-COPD matched controls, a higher incidence of hypertension, malignancy, diabetes mellitus, heart failure and peptic ulcer disease was observed among patients with COPD. In addition, the presence of COPD was associated with an increased odds of developing two or more comorbidities during the 5-year follow-up period. Taken together, our findings provide a broader view of the association between COPD and comorbidities and suggest that COPD per se exaggerates the risk of developing comorbidities. The information is practically useful in the evaluation and management of comorbidities in the COPD patients.

The novel information provided herein is to delineate the time course of pertinent comorbidities following a diagnosis of COPD. Since those comorbidities are prognostically associated with COPD, the messages, from a broad viewpoint, may inform guideline developers and health policy makers to propose an ideal strategy for comorbidity screening among patients with COPD. Moreover, from the individual viewpoint, the health care providers may be able to deliver better care of patients via improved understanding of comorbidity trajectories in COPD and timely identification and management of these comorbidities. Despite that it remains unsolved whether routine screening for comorbid conditions in COPD contributes to a better clinical outcome^5,21^, our study detailing the year-to-year change in the prevalence of comorbidities would certainly broaden our horizons for dealing with COPD from a longitudinal and whole-patient perspective.

As COPD becomes more and more understood to be a systemic inflammatory disease^22^, the view of COPD itself as an important etiologic factor for comorbidities has been an area of intense investigations. Several pieces of evidence demonstrate that the presence of COPD is associated with an increased risk of developing a number of comorbidities^3–5^. However, from another perspective, the concept of multimorbidity, i.e., the co-occurrence of multiple chronic diseases and medical conditions within one person, in the management of COPD has been insightfully studied in several works, indicating that COPD may just be one of a cluster of multiple conditions resulting from common risk factors^8,23,24^. Compared to other studies^25,26^, the present work is unique in terms of the matching strategy. Besides age, sex and smoking status, the non-COPD controls were matched to the COPD subjects for their comorbidities using the CCI. In this case, the COPD and non-COPD patients had similar baseline comorbidity burden and it would be more reasonable and appropriate to compare the change in comorbidities between the two groups of patients. Along this line, the prevalence of most comorbidities of interest at baseline was lower in our COPD cohort than in the controls since COPD was also taken into account while calculating the CCI. Nonetheless, the trend of change in the CCI and in the prevalence of certain comorbidities significantly varied between COPD and non-COPD control patients, highlighting and supporting the pathogenic role of COPD in the development of comorbidities, although the mechanistic links between COPD and its comorbidities remain to be elucidated.

The baseline prevalence of comorbidities in our COPD patients was in general comparable to that reported in previous studies^3–5,8^; however, our findings added to the existing knowledge by showing their evolution for the subsequent years. It is not surprising that in this aged population comprised of smokers, either current or former, a growing burden of comorbidities was evidently observed. Of those, the incidence of hypertension, malignancy, diabetes mellitus and peptic ulcer disease were significantly higher in patients with COPD than in non-COPD matched controls, which were consistent with prior literature^26–29^. However, the increased risk of heart failure, as seen in our COPD cohort, was not proven in other population studies^30,31^. On the contrary, it has been shown that COPD is associated with a higher incidence of coronary artery disease and atrial fibrillation^32–34^, but the present study did not disclose such a relationship. The discrepancies may be attributable to one or more factors, such as the study design, case definition and ascertainment, control selection and outcome of interest. No matter what, in view of the interaction of complex networks between COPD and its comorbidities, our study results reinforce the need for a multidisciplinary approach for taking care of COPD patients.

This study has limitations that may limit its generalizability to other COPD populations. First, patients with COPD who lost to follow-up or died during the follow-up period were excluded, so our prevalence and annual incidence of comorbidities are likely to over- or under-estimate the true figures for the entire population with COPD. Second, the proportion of COPD cases who were male (89%) was higher than that seen in most COPD studies^12,35,36^, which may represent a selection bias. However, the aforementioned limitations would not have influenced the differences that we observed between COPD and matched control patients, and therefore may not affect our main findings and conclusions. Third, chronic bronchitis, another smoking-related respiratory disease, may cause systemic inflammation, and is associated with worse clinical outcomes of COPD patients and with an increased risk of other comorbidities^37^. Since various definitions of chronic bronchitis have been used in the literature and clinical practice^37^, we are unable to ascertain its prevalence in both groups of our population. Thus, the impact of chronic bronchitis on our study findings is uncertain and this limitation should be born in mind. Fourth, changing in smoking status and tobacco consumption may also affect the symptoms and lung function in COPD patients and the development of smoking-related comorbidities in smokers^38,39^. However, given the retrospective design of the study, we could not accurately retrieve the information about the amounts of tobacco consumption and smoking cessation efforts from the medical records of our study population. Thus, these confounders should be considered when interpreting the study results. Last, without systematic diagnostic tests, certain comorbidities, such as coronary artery disease and malignancy, may go undiagnosed at baseline. Nonetheless, this issue should be present in both groups of patients and does not significantly affect our study findings.

In summary, this study provides a more comprehensive view of COPD by showing 5-year trajectories of its pertinent comorbidities. The findings suggest that an increasing comorbidity burden is an unavoidable issue in patients with COPD, and a systematic and holistic approach is often needed to take care of this patient population. In addition, the presence of COPD may be considered a pathogenic factor involved in the development of its comorbidities. Further studies are required to explore the potential benefits and roles of proactive screening of comorbidities in the COPD population.

## Data Availability

The datasets used and/or analysed during the current study are available from the corresponding author on reasonable request.
